# Anticipative QoS Control: A Self-Reconfigurable On-Chip Communication

**DOI:** 10.3390/mi13101669

**Published:** 2022-10-04

**Authors:** Wen-Chung Tsai, Hsiao-En Lin, Ying-Cherng Lan, Sao-Jie Chen

**Affiliations:** 1 Department of Intelligent Production Engineering, National Taichung University of Science and Technology, Taichung 404336, Taiwan; 2 Marvell Technology Group, Taipei 114740, Taiwan; 3 Department of Electrical Engineering, National Taiwan University, Taipei 106216, Taiwan

**Keywords:** anticipative control, bidirectional-channel, network-on-chip, on-chip communication, quality-of-service, self-reconfigurable

## Abstract

A self-reconfigurable Network-on-Chip (NoC) architecture that supports anticipative Quality of Service (QoS) control with penetrative switch ability is proposed to enhance the performance of bidirectional-channel NoC communication while supporting prioritized packet transmission services. The anticipative QoS control not only allows each communication channel to be dynamically self-configured to transmit flits in either direction for a better channel utilization of on-chip hardware resources, but also enhances the latency performance for QoS services. The proposed anticipative control is based on penetratingly observing channel direction requests of routers that is two hops away from the current one. The added ability enables a router to allocate high-priority packets to a dedicated virtual channel and then rapidly bypass it to the next destination router. The provided flexibility of packet switch promises better channel bandwidth utilization, lower packet delivery latency, and furthermore guarantees the high-priority packets being served with a better QoS. Accordingly, in this paper, an enhanced NoC architecture supporting the hybrid anticipative QoS, penetrative switch, and bidirectional-channel control, namely Anticipative QoS Bidirectional-channel NoC (AQ-BiNoC) is presented. Tested with cycle-accurate synthetic traffic patterns, significant performance enhancement has been observed when the proposed AQ-BiNoC architecture is compared against conventional NoC designs.

## 1. Introduction

Increasing transistor density, higher and higher operating frequency, and shorter product life cycles characterize the semiconductor industry trend. Under these conditions, designers are developing Integrated Circuits (ICs) that integrate complex heterogeneous functional elements into a single chip, known as a System-on-Chip (SoC), which is composed of many Intellectual Property (IP) cores on a semiconductor chip. In an SoC, such IP cores must be connected with an interconnection mechanism known as bus architecture, which includes physical interfaces and connection wires, to enable communications among the SoC components. The bus architecture has successfully been used in conventional SoC designs [1]. However, there exist some problems when the SoC system scale increases [2]. For example, there are tens, or hundreds of cores in an SoC chip, and the interconnection distance and electric resistance can require multiple clock cycles for a communication pair between two cores in the SoC chip. To meet the need for new generation system designs, Network-on-Chip (NoC) [3,4] was proposed and had continued to be the focus of all people who work to enhance the performance of on-chip communications [5,6,7,8,9,10].

Furthermore, in a high-end system, as network complexity grows rapidly, the interconnection performance between cores becomes more important. Accordingly, in some applications of interconnecting networks, it is useful to divide network traffic into several prioritized classes to efficiently manage the resource allocations to different packets [11]. Recently, Lu et al. [12] provided a Quality of Service (QoS) analysis methodology for executable micro-architectural specification (i.e., NoC), in which elementary flow delivery under resource control can be precisely modeled. Rahmati et al. [13] realized a simple prioritized arbitration to differentiate high and low-priority traffic flows, consequently there was substantial performance improvement for high-priority flows. Wang et al. [9] designed a QoS provisioning NoC architecture, which is an inheritance mechanism to convert QoS information into the request buffers of the NoC.

A basic prioritized service gives packets of higher class a strict priority in the allocation of buffers and channels over packets of a lower class. Traffic classes fall into two broad categories: Guaranteed Service (GS) and Best Effort (BE) as applied in [13]. GS classes are guaranteed a certain level of performance. In contrast to GS, the network makes no strong promise about BE packets. Depending on the network, BE packets may have arbitrary delays or even be dropped. The network will simply make the best effort to deliver the packet to its destination. In general computer networks, there are various algorithms targeting the performance between GS and BE traffics. However, not every algorithm can fit in the NoC architecture because there are many differences between computer networks and network-on-chip. The state-of-the-art QoS mechanism for NoC can be categorized into connection-oriented and connection-less [11]. In the connection-oriented scheme, the connection path between source and destination is built and preset before the packets are injected into the network. It is reliable to satisfy the QoS requirement, but comes with great hardware cost because the resource reservation and allocation take huge control and storage elements. For connection-less schemes, reconfigurable control schemes are employed in existing NoC architectures. For example, Bondade et al. [14] provided a self-reconfigurable channel data buffering scheme while operating at optimal power levels. Nain et al. [8] realized an adaptive routing algorithm for demanding latency and throughput for fault-tolerant NoCs. Wang et al. [15] proposed a ring-based interconnect architecture that can globally reconfigure its NoC rings with high flexibility in adapting to the traffic flows. Mandal et al. [16] generated a latency-optimized reconfigurable NoC architecture that is customized for Deep Neural Networks (DNNs).

Additionally, a cognitive communication system [17] that is cognizant of rapidly changing on-chip traffic requirements promises great performance enhancement [18]. For example, Melo et al. [5] analyzed the router behavior for detecting signal upset to minimize error propagations, and Tang et al. [19] implemented a congestion avoidance method for NoC, based on the speculative reservation of network resources. Recently, Mehranzadeh et al. [6] designed a congestion-aware output selection strategy based on calculations of congestion levels of neighboring nodes, and Giroudot et al. [7] realized a buffer-aware worst-case timing analysis of wormhole routers with different buffer sizes when consecutive-packet queuing occurs. Especially, a network-cognitive traffic control mechanism for integrated congestion control and flow control was proposed in [20].

Except for the uni-directional NoC architectures mentioned above such as [14,20], Bidirectional-channel NoC (BiNoC) [21] is an on-chip channel direction self-reconfigurable network design, which allows the channel to be dynamically configured to transmit data in either direction, therefore BiNoC can achieve a better network performance due to its better channel utilization. FSNoC [22] is a flit-level speedup scheme to improve the NoC performance using self-reconfigurable bidirectional channels as well as [21]. Furthermore, FSNoC allows flits within the same packet to be transmitted simultaneously, that is for an input buffer, which can support reading and writing two flits at the same time. In conventional NoCs, a router uses a pair of unidirectional channels to connect with a neighbor router. One of the channels is used for transmission (TX) and the other is reserved for reception (RX). Compared with the unidirectional channels, bidirectional channels allow being dynamically configured to transmit data in either direction. By the self-reconfigurable channel direction provided, BiNoC [21] and FSNoC [22] can achieve better network performance due to their better channel utilization.

To support QoS in the conventional BiNoC architecture [21,22,23], a key challenge is that performance balance between GS and BE traffics is required [13], because the router resource grantee is set based on the speculation that the path is not used by any other higher priority packet. When a channel direction is monopolized by only one packet for a long time, it will make other packets stalled or delayed. With just sacrificing the performance of BE traffics, the QoS performance improvement to the GS packets still become saturated soon. Accordingly, we propose an Anticipative QoS-controlled BiNoC router architecture to further improve the performance of GS packets. The main idea is to reduce transmission latency and inefficient resource occupation. Better bandwidth utilization can be obtained from the bi-direction channel switch and Virtual Channel (VC) [24,25] prioritized authority. Moreover, the balance control between the improvement and scarification of packets is established by a provided calculation scheme as introduced in the following sections.

This paper is organized as follows: in Section 2, the background of typical NoC routers and the characteristic and architecture of QoS-aware BiNoC is explained. In Section 3, the design methodology, including the proposed Anticipative QoS-controlled Bi-directional NoC (AQ-BiNoC), is introduced. In Section 4, the experimental results with performance analyses and discussions are presented, and a conclusion is drawn in Section 5.

## 2. Background

In this section, we will briefly review some concepts on the design of NoC and BiNoC router architectures, a popular flow control method using virtual channels, and the QoS mechanisms implemented in BiNoC which provide both guarantee service (GS) and best effort (BE) traffic classes. Several distinguished works in these research topics will be noted and introduced.

### 2.1. Network-on-Chip Architecture

**Figure 1:**
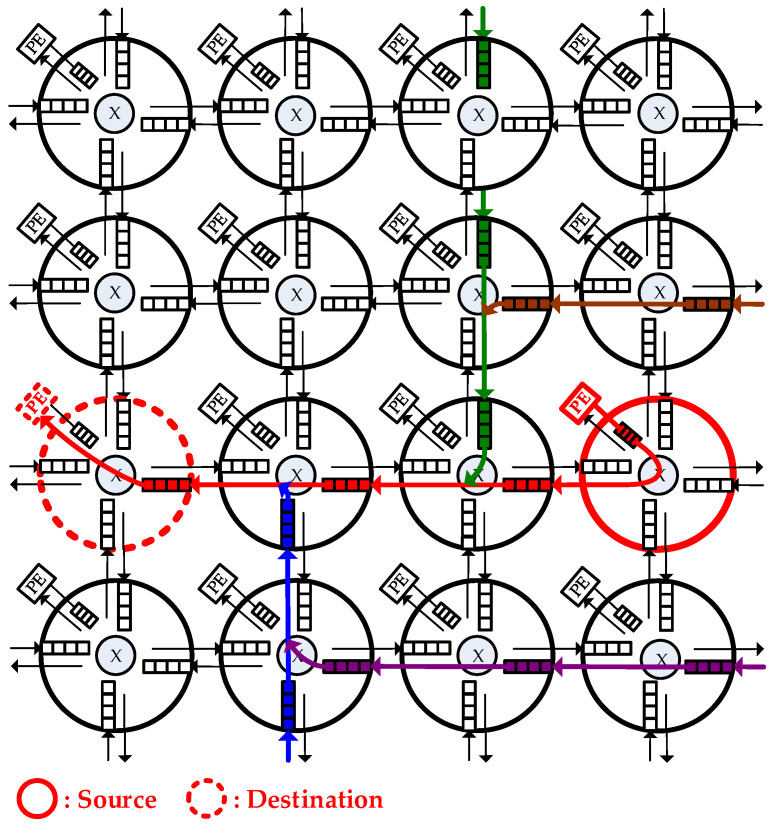
A 2-D mesh NoC architecture.

The NoC [3,4] architecture specifies the network topology and physical organization of an interconnection network. Like all digital system designs, we should determine the constraints and build different solutions to the problems that we want to solve [26,27,28], two-dimensional (2-D) mesh is the most popular topology for current NoC design because of its simplicity and regularity. As Figure 1 shows, In the NoC, each router buffer is connected to a Processing Element (PE) or several neighbor routers, and each PE can be an IP core such as a microprocessor.

Besides, an NoC consists of physical channels that comprise the communication architecture, the channels are sets of wires which connect each pair of neighbor routers to enable the packets to be delivered through routers. Figure 1 shows a typical NoC architecture in a 2-D 4 × 4 mesh network. In this, when a message is sent from a source PE (marked as red), it is encapsulated into a packet and a packet is composed of several flits (flow control digits). The packet is delivered by several routers until it reaches the destination PE (marked as red). Last, the destination PE unpacks it into the primitive message. For every packet entering a router, data flits of the packet will be written into the input buffer of the port where it is entered. Additionally, the control logic will detect its routing path and the channel which it should head for. After winning arbitration (as flits marked as red), the data flits can leave the router from the output port whose direction is decided from the routing information. Otherwise, the data flits (as flits marked as other colors) will be stored in the buffer until it is allowed to leave by later arbitration.

### 2.2. Pipeline Execution of Router

**Figure 2:**
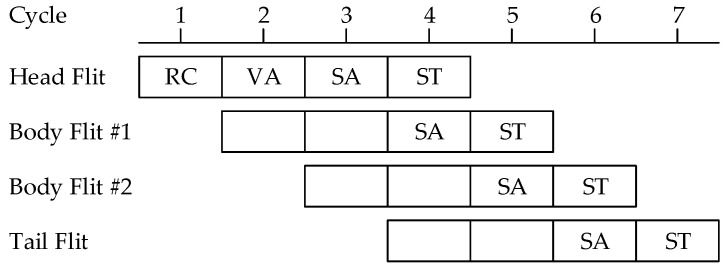
Pipeline stages of packet traversals in a router.

Figure 2 shows the pipeline stages for switching a packet in a router. Each flit of a packet proceeds through the stages of Routing Computation (RC), Virtual channel Allocation (VA), Switch Allocation (SA), and Switch Traversal (ST) [29]. The RC and VA stages are only performed for the head flit. Body flits passing through these control stages do not need the RC and VA stages. The SA and ST stages operate on every flit of the packet.

### 2.3. QoS-Aware BiNoC

**Figure 3:**
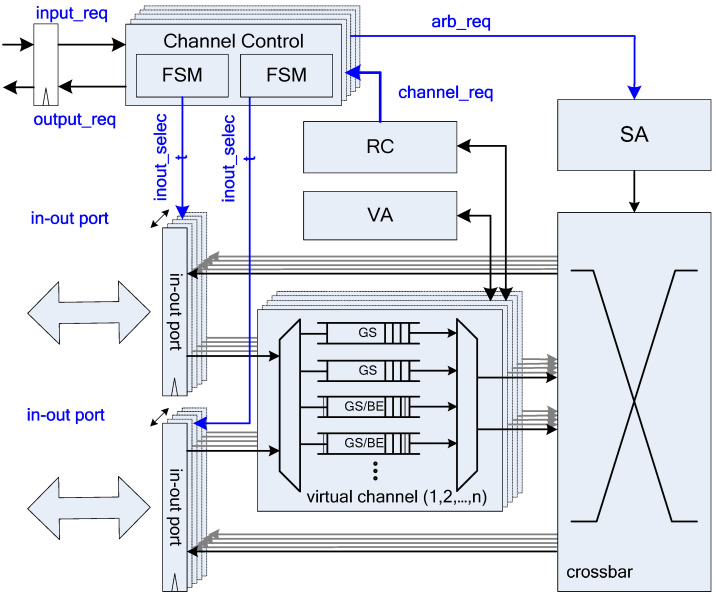
QoS-aware BiNoC architecture.

Typical NoC owns channels with fixed directions. It is observed that under various traffics, one channel may be full of traffic and another is empty and idle. The basic concept of BiNoC [23] is switching channel direction to achieve better bandwidth utilization. However, BiNoC architecture only provides good latency results for BE traffic because of its channel utilization flexibility, but it is incapable of supporting critical communication guarantees that are much more important for real-world applications. Figure 3 shows the basic architecture of a QoS-aware BiNoC router [30]. There are four virtual channels in each input port. The main concepts of the QoS-aware BiNoC are described in the following sub-sections.

### 2.4. Bi-Directional Channel Routing Direction Control

**Figure 4:**
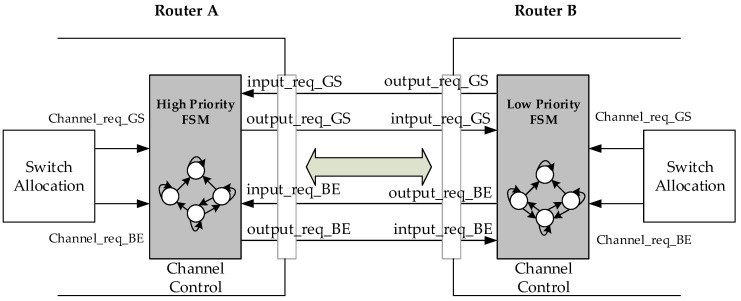
Inter-router transmission control.

Figure 4 shows that the BiNoC inter-router transmission control includes a Finite State Machine (FSM) and two input/output signals on each side. The FSMs are driven by the SA stage needing and another side’s requests. The output_req_BE/GS signal rises when a BE/GS packet at the SA stage requests the direction. Oppositely, the other side will receive the corresponding input_req_BE/GS signal to the FSM.

**Figure 5:**
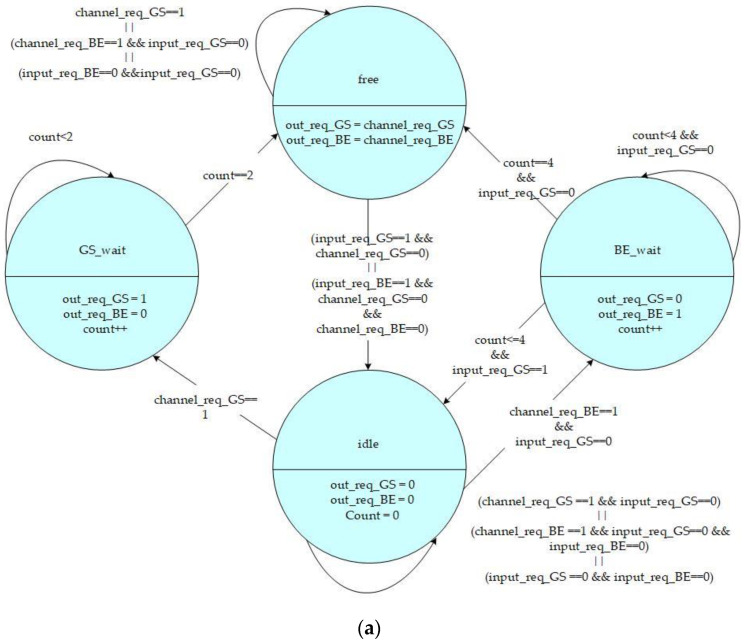

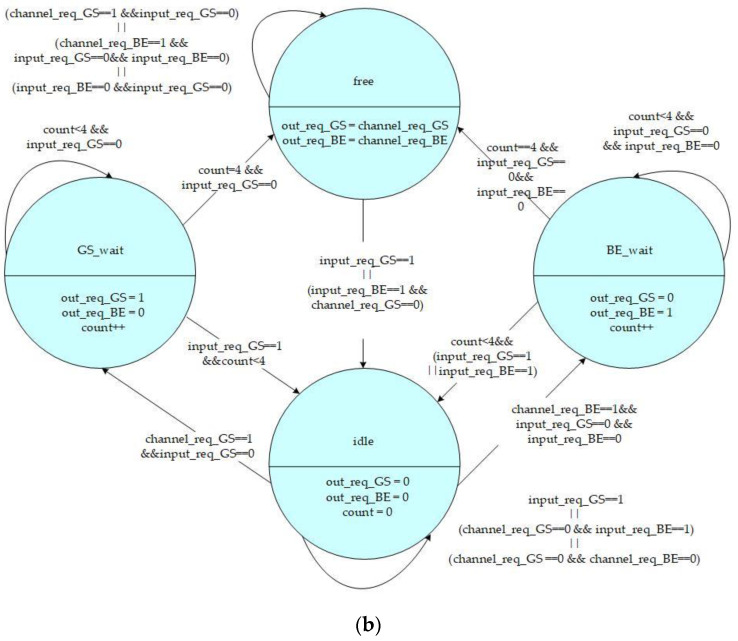
(**a**) High-priority and (**b**) low-priority FSMs used in QoS-aware BiNoC.

The states and control signals of the high-priority and low-priority channel control FSMs are shown in Figure 5a,b, respectively. The FSM includes four states: *Free*, *GS_wait*, *BE_wait*, and *Idle* which are defined as follows:

- *Free* State: The channel is ready to output data to another connection router.
- *GS_wait* State: An intermediate state for the GS packet to transform from the *Idle* state to a *Free* state. It is a temporary state for the channel to change its direction from input to output.
- *BE_wait* State: An intermediate state for BE packet to transform from the *Idle* state to a *Free* state. It is also another temporary state for the channel to change its direction from input to output.
- *Idle* State: The channel is available to input data from another connection router.

When the state of FSM is idle, it will remain at this state only if there are no data needed to transmit outbound, or the input request from another connection router has a higher priority than the local channel output request. Moreover, when the state of FSM is idle, as soon as a GS channel request from the input unit is received, the state will be triggered into a *GS_wait* state. Additionally, if a BE channel request is received without any GS request triggering, the state will change into a *BE_wait* state.

### 2.5. Problem of Existing BiNoC Design

**Figure 6:**
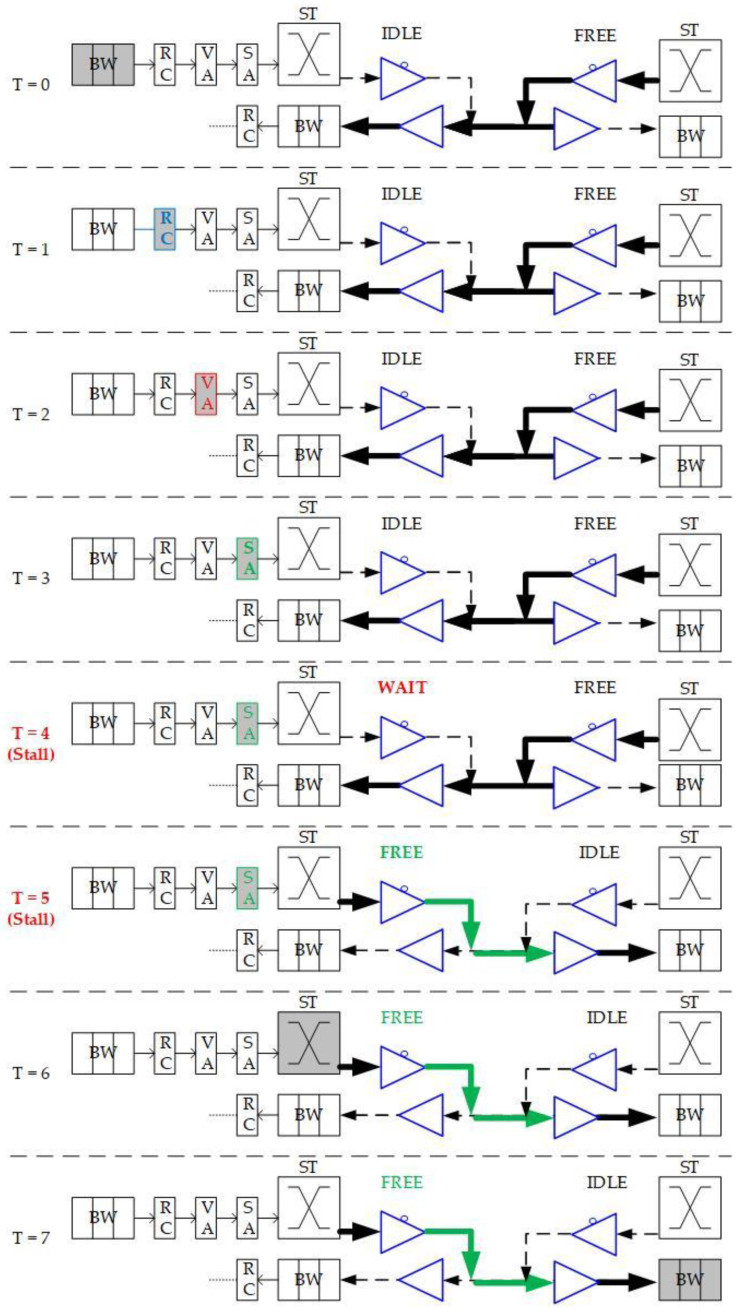
Packet traversals in pipeline stages and channel direction changes.

As a self-configurable and scalable approach for high-performance and energy-efficient NoC computing systems [10], BiNoC is a channel direction self-configurable network design. By the channel flexibility provided, it takes less overhead and achieves better network performance and channel utilization. Using this concept, BiNoC explores a similar idea as a mechanism to decrease the intermittent traffic congestion in an NoC communication backbone, and hence enhance its overall performance. However, there is still a problem with direction-switched BiNoC. As shown in Figure 6, the waiting stage causes an idle channel with additional time-consumption for direction switching. The pipeline stage executes Buffer Writing (BW) at time slot 0 (T = 0), Routing Computation (RC) at T = 1, Virtual Allocation (VA) at T = 2, and Switch Allocation (SA) at T = 3. However, when the direction is detected as reversed at the SA stage, additional cycles (e.g., T = 4 and T = 5) are needed to wait for the channel to switch to the side that the packet requests. The wait state of FSM is set because there is a propagation delay between two routers. For example, as shown in Figure 6 at T = 4, there may be data on another side being sent to the local router. If we just turn the channel direction reversely, the data will be lost, since the tri-state buffer acts in one direction at one time. This waiting behavior results in stalls (i.e., T = 4 and T = 5) in the pipeline and is inefficient for the transmission performance, even if there are no data needed to transmit from the neighbor router.

To eliminate the time in waiting for the direction switch, we will move the channel direction request mechanism forward to the routing stage, because the RC stage is the first stage that provides the output direction information of packets. As illustrated in Figure 6, the original channel direction request based on the VA and SA allocations is successful because the channel authority is granted based on the buffer (VA) and channel (SA). The anticipative channel direction control introduced in Section 3.2 can be used to request direction authority even when the VA/SA stage status is unknown. Additionally, the request sending is speculating that the VA/SA request will be granted and successfully passed. This work will be mounted with GS packets only since the GS packets have more possibility to grant both VA/SA.

**Figure 7:**
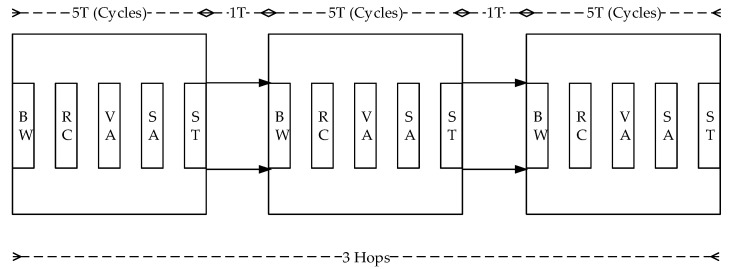
Five pipeline stages in a router and two hops to the destination.

Another phenomenon is illustrated in Figure 7. In every router, there are five stages of BW, RC, VA, SA, and ST stages to proceed. Besides, there are two hops to transmit among routers, then it causes 3 × 5 + 2 = 17 cycles to propagate the packet from the source router to the destination two hops away. 

However, the actual pipeline stages count usually can be more than the pipeline stages because of the stall. In a QoS scheme, there is a unique characteristic in the GS packet traversal, that is, the GS packet usually grants the allocation competition in a routing path. It represents that most of the allocation pipeline stages such as Switch Allocation or Virtual Channel Allocation are a waste of time because the GS packets have a must-winning competition. As a result, we try to reduce these ignorable stage cycles to decrease packet latency. The proposed design methodology is introduced in the following sections.

## 3. Design Methodology

**Figure 8:**
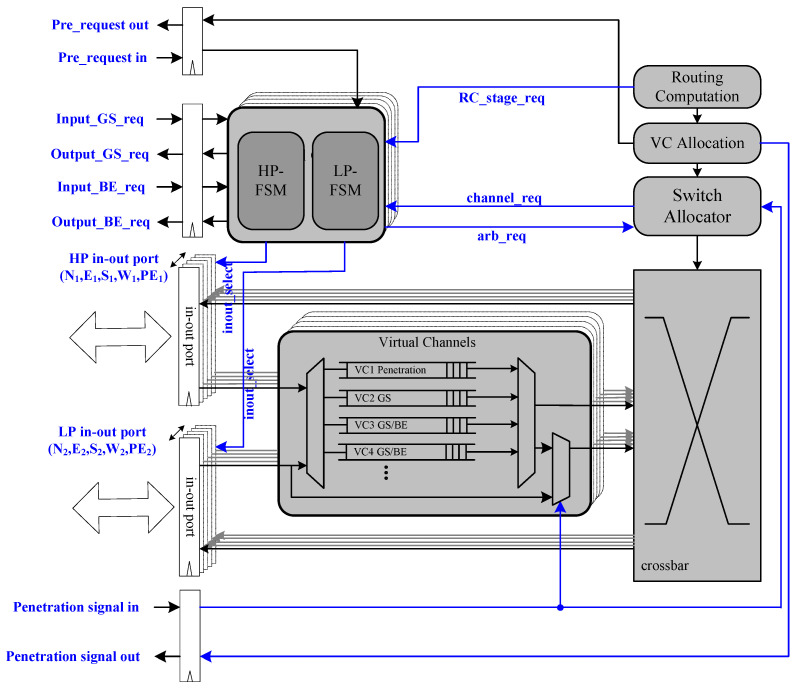
Anticipative QoS-controlled BiNoC router architecture.

Based on the BiNoC architecture introduced in Section 2 and combing the idea discussed in the last section, we adopt the bi-direction channel transmission scheme with QoS support and present a practical router architecture to be implemented with a virtual channel in a pipeline fashion. Figure 8 shows the Anticipative QoS-controlled BiNoC router architecture.

### 3.1. Router Implementation

**Figure 9:**
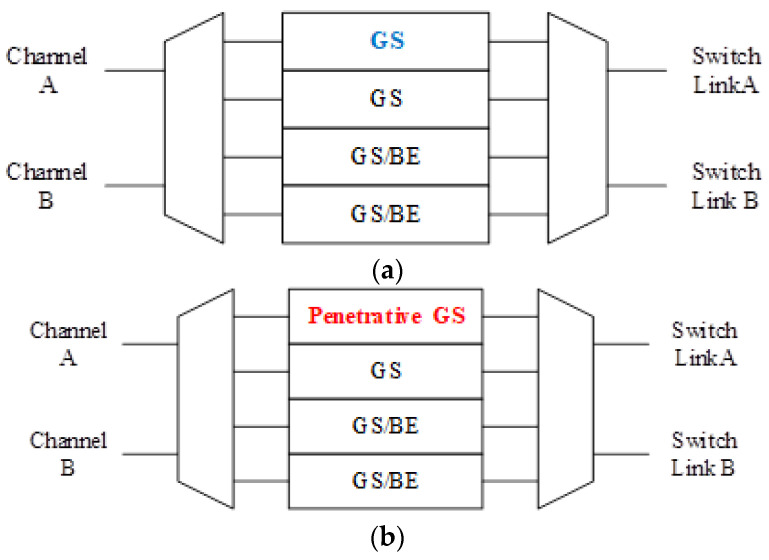
Virtual channel architecture comparisons between (**a**) the original and (**b**) the modification.

The basic router architecture as shown in Figure 8 is simple and inherits all the characteristics of the QoS-aware BiNoC. The virtual channel architecture is developed from the prioritized virtual channel, but the priority level is different from the original one. As Figure 9a shows, the original virtual channel priority is divided into GS and GS/BE levels. In our design as shown in Figure 9b, we separate the four virtual channels into one Penetrative GS channel, one GS channel, and two GS/BE channels. The additional bypass function will be mentioned in Section 3.3. The penetrative GS channel receives the packets sent from the router, which is at a distance of two hops away instead of the neighbor routers.

### 3.2. Anticipative Bidirectional-Channel Control

**Figure 10:**
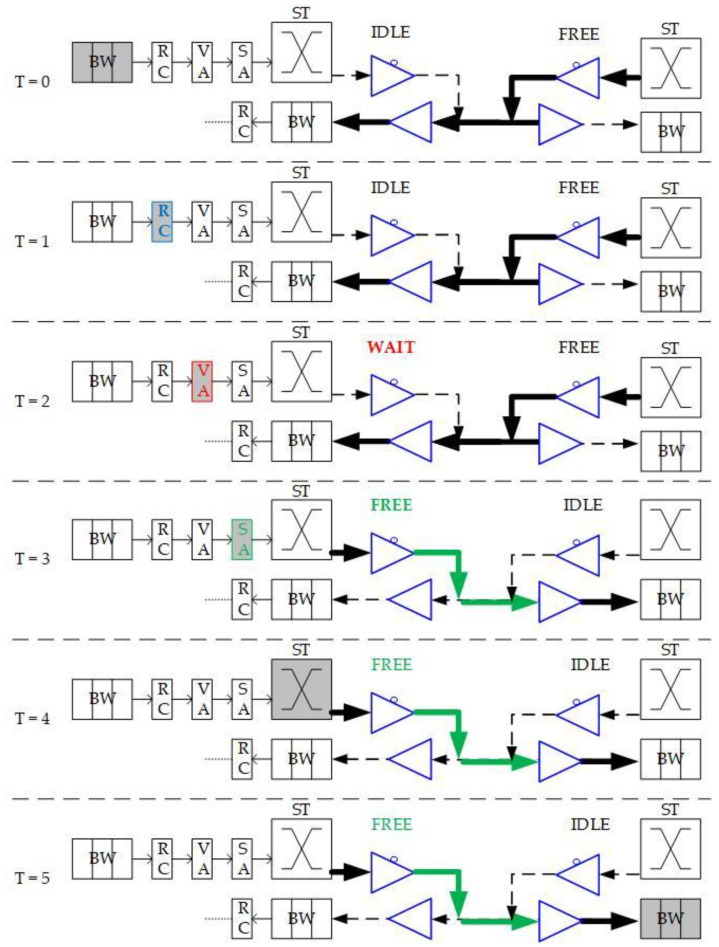
Optimized packet traversals in pipeline stages and channel direction changes.

In this section, we will mention how to previously request changing channel direction using speculation for the self-reconfigurable bidirectional-channel control. In Figure 8, we can see the request from the RC stage to the channel controller. This implies that the request will be directly sent to the channel controller when the routing computation detects that the packet has a high-priority on being granted in other stages. However, we may not grant all packets’ request directions that they are heading for. In the pure pre-request experiment, namely Pre-request BiNoC (Pre-Req BiNoC), discovering every request of direction by a packet at the routing stage causes worse performance, because most packets may change direction successfully but fail in switch allocation or virtual channel allocation. Therefore, we need to limit the number of packets that are available to use a speculative switch. The anticipative bidirectional-channel control is suitable only for GS packets. As Figure 10 shows, the previous request at the RC stage brings effective time reduction, compared to that in Figure 6.

### 3.3. Penetrative Switch Ability

**Figure 11:**
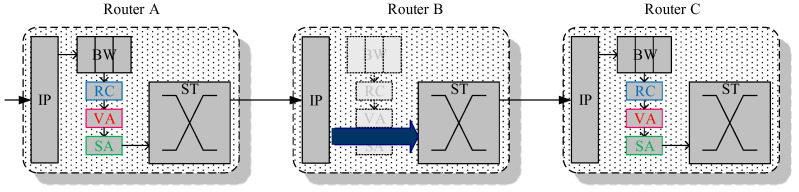
Penetration execution among routers.

In this section, we will introduce how to make GS packets self-reconfigurable to bypass the router’s pipeline. Figure 11 shows the condition of whether a packet can bypass the router or not as follows. A GS packet in Router A is bypassing Router B and then transmitted into Router C. The packet first receives penetration availability information from the routing computation at the RC stage of Router A. It then requests the destination virtual channel buffer of Router C at the VA stage of Router A. Finally, after switch allocation, it can transmit the output data from Router A with an additional penetration signal. This signal can stall the original SA process to the same direction as Router B and set the crossbar to a specified output direction. The packet can directly traverse the ST stage without additional allocation. After departing Router B, the packet arrives at Router C and will follow the same steps of normal router pipelining to complete its switches.

**Figure 12:**
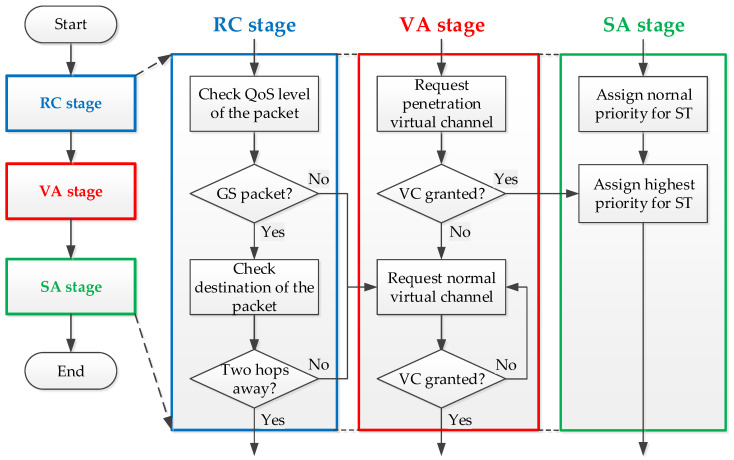
Penetration execution in a router.

To further introduce the execution sequence of packet penetration, we provide a flow diagram in Figure 12. If there is a piece of available information detected in the RC stage, the packet having penetration conditions will compete for the destination virtual channel at the VA stage, and the others will perform normal virtual channel allocation. After having the penetrative virtual channel granted, the packet will have a higher priority for competition at the SA stage. The packet which has the authority to penetrate the router will raise the signal to stall the router at the next hop.

### 3.4. Proposed Anticipative QoS-Controlled BiNoC

**Figure 13:**
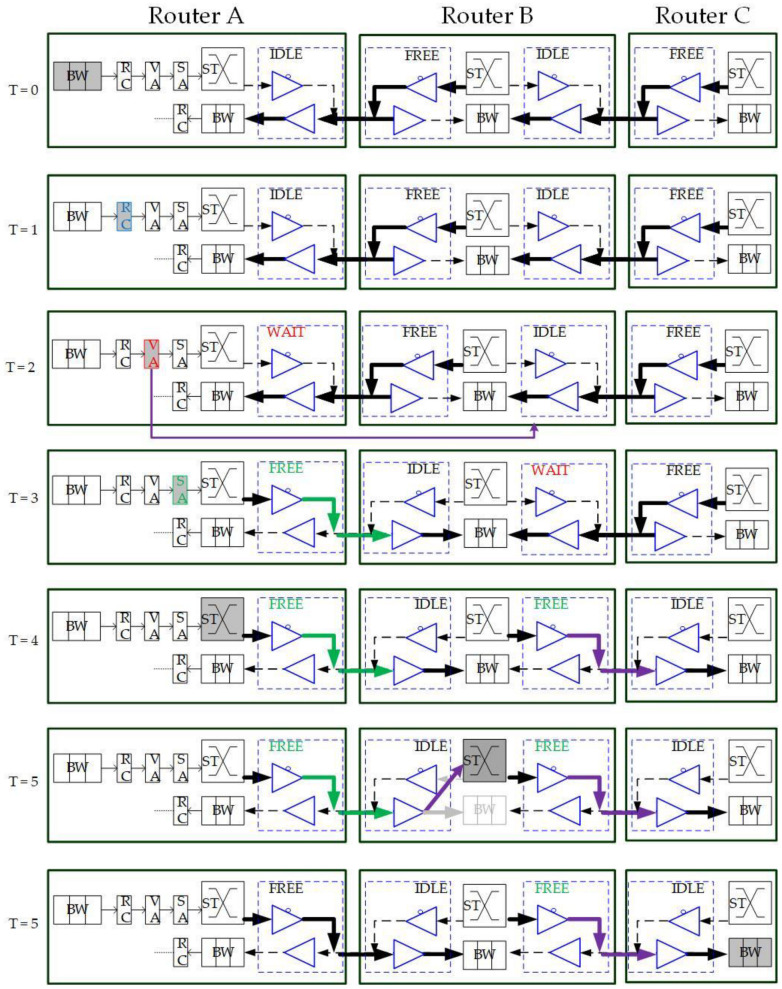
Packet traversals and channel direction changes of the proposed AQ-BiNoC.

To integrate the above two specific abilities with BiNoC, we propose an improved QoS-aware BiNoC with anticipative bidirectional-channel control and penetrative switch ability, namely AQ-BiNoC. The channel direction control becomes more complex because the output direction of the bypassed router has to be set to the designed direction. Figure 13 illustrates an execution example of packet traversals and direction changes among three routers. At T = 2, when the packet is allocated to the penetrative virtual channel, the channel direction request of the next hop from Router B will be sent. At T = 4, the channel direction between Routers B and C is from B to C. At T = 5, the packet will bypass the router pipeline and directly use the crossbar with configuration to traverse through Router B to reduce transmission latency. Related experimental results with performance analyses and discussions are presented in the following sections.

## 4. Experiment Result and Discussion

To make a performance comparison and hardware cost estimation between the QoS-aware BiNoC and our proposed AQ-BiNoC, a cycle-accurate simulation environment was implemented with different router architecture designs in Verilog hardware description language. Every design is comprised of multiple functional blocks including routing, channel control, arbitration, and switch fabric. The traffic patterns in our simulation environment are synthetic traffics [31] with varying QoS utilization from 5% to 50%. The environment simulates wormhole switching with a packet separated into the head, body, and tail flits. The applied routing algorithm is XY [32] which routes packets X direction first and then Y direction. Besides, for packets with the same QoS level, a round-robin rule [13] is applied to deal with related resource allocation problems. 

### 4.1. Performance Evaluations among Different NoC Routers with Different Traffic Patterns

Performance evaluations among different NoC routers of Typical-NoC, BiNoC, Pre-request BiNoC (Pre-Req BiNoC), and Anticipative QoS BiNoC (AQ-BiNoC) are implemented in our experiments. To make fair comparisons, all four routers use similar configurations as shown in Table 1.

In synthetic traffic analysis, the physical layer of our simulation environment comprises 8 × 8 nodes connected as a mesh array. Each packet has a constant length of 16 flits. The tests performed sent packets at varying injection rates (from 0.032 to 0.8 flit/node/cycle) and varying GS packet percentages (from 0.01 to 0.5) under different traffic for 25,000 cycles. Four types of synthetic traffic patterns were run: uniform, regional, transpose, and hotspot. In uniform traffic, a node will be the destination of every other node with equal probability based on the injection rate. In regional, 90% of the packets are sent to the destination at distance of less than three hops, and most transmissions occur among two neighbor routers. In transpose traffic, a node at coordinate (*i*,*j*) will send a packet to the destination whose coordinate is (*j*,*i*). In hotspot traffic, most packets are sent to the same destination at once. The traffic is similar to real-case traffic because in SoC most IP cores have communication with the main PE (i.e., processor). To evaluate the QoS performance, a value of GS packet percentage is defined as the count of GS packets over the count of total packets, thus the GS packet percentage reveals the bandwidth utilization of high-priority packets in the performance simulation for specific NoC router architecture.

### 4.2. Experiments with Uniform Traffics

**Figure 14:**
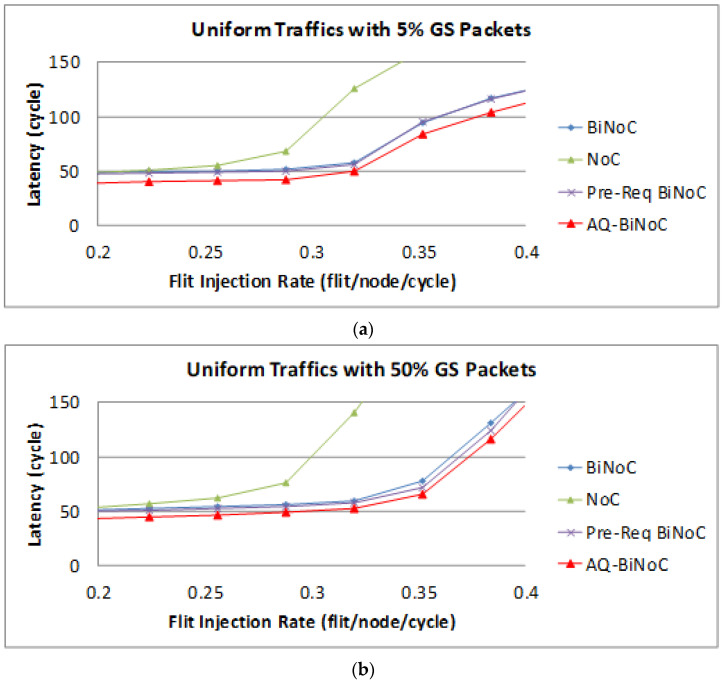
Packet latency analyses at (**a**) 5% and (**b**) 50% GS packet percentage under Uniform Traffic.

We analyze the simulation results obtained from low GS packet percentage first under the uniform traffic. Figure 14a,b represent the GS packet latency at varying injection rates (from 0.032 to 0.416) with GS packet percentages of 5% (low) and 50% (high), respectively. In Figure 14a, we can discover that the average latency of AQ-BiNoC is lower than BiNoC, Pre-Req BiNoC, and traditional NoC in percentages of 14.95%, 13.80%, and 35.12%, respectively. Figure 14b shows the latency of uniform traffic under a higher GS packet percentage of 50%, AQ-BiNoC is still overcome other compared NoC Architectures. The average latency of AQ-BiNoC is lower than BiNoC, Pre-Req BiNoC, and traditional NoC in percentages of 12.57%, 11.05%, and 41.63%, respectively. Besides, the simple Pre-Req BiNoC still has a similar latency curve compared with BiNoC. The minor improvement comes from the inter-router arbitration mechanism.

**Figure 15:**
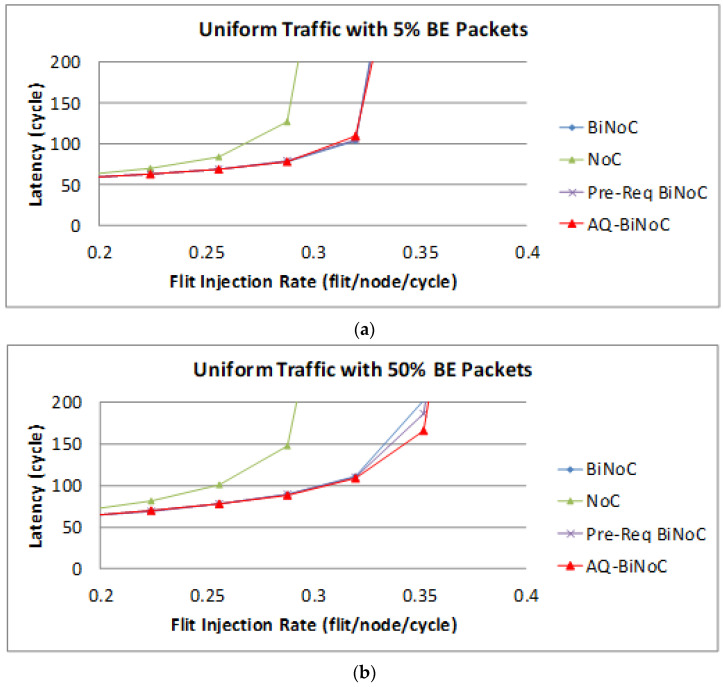
Packet latency analyses at (**a**) 5% and (**b**) 50% BE percentage under Uniform Traffic.

The following Figure 15 shows the BE packet latencies in different QoS percentages over different injection rates. In Figure 15a, we can discover that the average latency of AQ-BiNoC is lower than BiNoC, Pre-Req BiNoC, and traditional NoC in percentages of 1.48%, 2.95%, and 55.44%, respectively. Figure 15b shows the latency of uniform traffic under a higher BE percentage of 50%, the average latency of AQ-BiNoC is lower than BiNoC, Pre-Req BiNoC, and traditional NoC in percentages of 9.18%, 8.84%, and 70.69%, respectively. We can notice that the latencies of BE packet in BiNoC, Pre-Req BiNoC, and AQ-BiNoC almost have no difference. The reason is that most functions implemented are used to serve GS packets only. When the GS packets travel very fast in the network, the resources occupied by GS packets could also be released as soon as possible. That is why in some cases the BE latency in AQ-BiNoC is better than the one in BiNoC.

**Figure 16:**
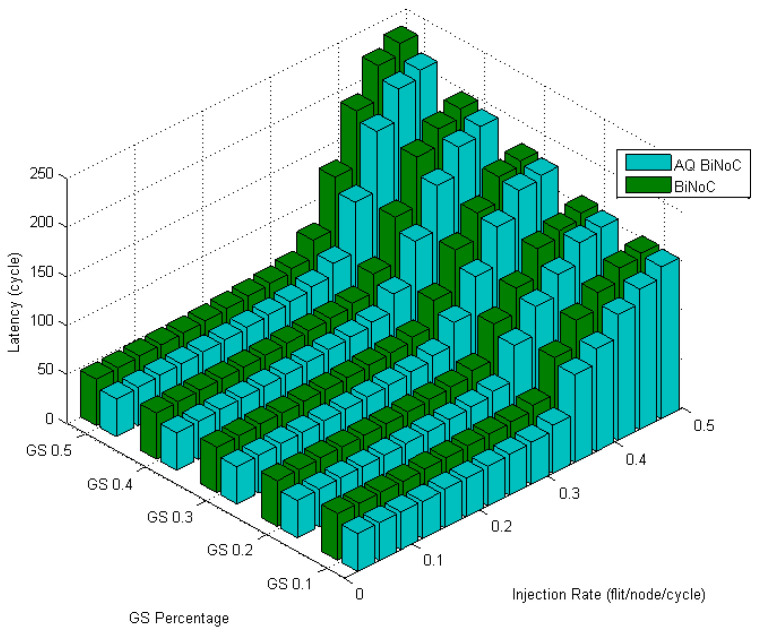
Latency of AQ-BiNoC compared with BiNoC in Uniform Traffic.

Figure 16 illustrates the latencies of AQ-BiNoC compared with BiNoC. Even under the condition where the GS packet percentage and the injection rate are high, we can observe that the AQ-BiNoC has better latency performance than BiNoC and NoC architectures under uniform traffic.

**Figure 17:**
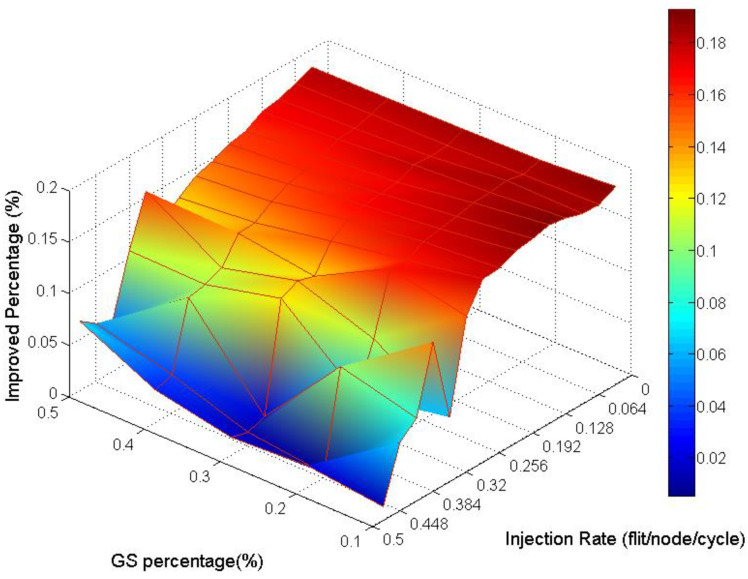
Performance improvement of AQ-BiNoC compared with BiNoC in Uniform Traffic.

Analyzing the overall performance improvement of uniform traffic as shown in Figure 17, we can find that under higher GS packet percentage, the improvement scale becomes smaller. It is reasonable because performance will become worse as the same resources such as penetrative virtual channels are shared by more requesters. But the improvement is still effective under a higher GS packet percentage in uniform traffic. This phenomenon can be realized by the characteristic of uniform traffic where the packet flows are separated into the whole network equally. Thus, the resources can be averagely shared to whole GS packets which need high transmission quality.

### 4.3. Experiments with Regional Traffics

**Figure 18:**
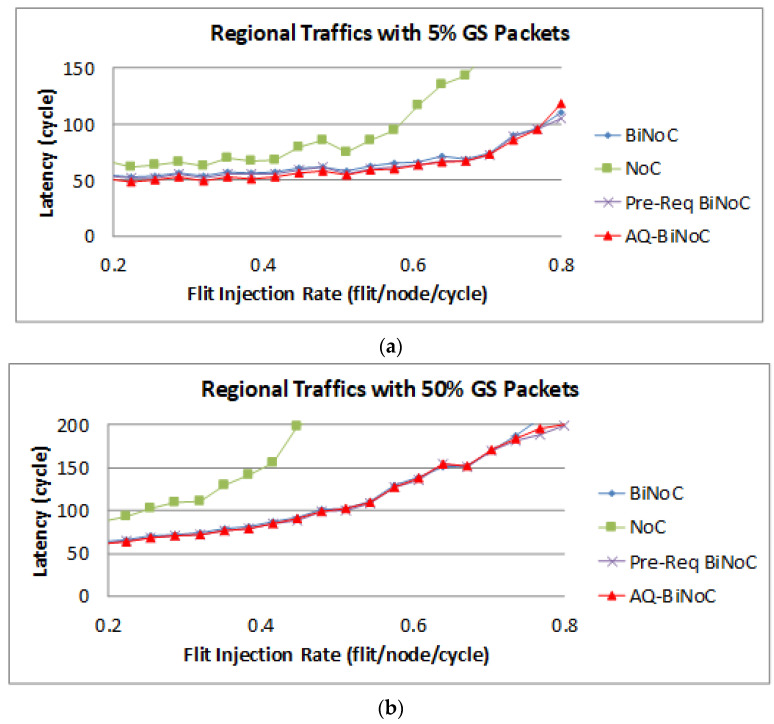
Packet latency analyses at (**a**) 5% and (**b**) 50% GS packet percentage under Regional Traffic.

Figure 18a,b represent the GS packet latency at varying injection rates (from 0.032 to 0.8) with GS packet percentages of 5% (low) and 50% (high), respectively. As Figure 18a shows, we can discover that the average latency of AQ-BiNoC is lower than BiNoC, Pre-Req BiNoC, and traditional NoC in percentages of 5.27%, 2.65%, and 36.15%, respectively. Figure 18b shows the latency of regional traffic under a higher GS packet percentage of 50%, the average latency of AQ-BiNoC is lower than BiNoC, Pre-Req BiNoC, and traditional NoC in percentages of 1.80%, −0.78%, and 53.57%, respectively. We can observe that the improvement of packet latency is not oblivious under regional traffic, the most important reason for the latency of each architecture being so close is that the regional traffic is transmitting data to a close destination mostly. The latency decrease is from the anticipative bi-direction channel control only in the case where there is no longer enough distance of packet transmitting. It causes the Pre-Req BiNoC and AQ-BiNoC to have similar performance curves. Additionally, they both improve the neighborhood transmission by the inter-router channel control.

**Figure 19:**
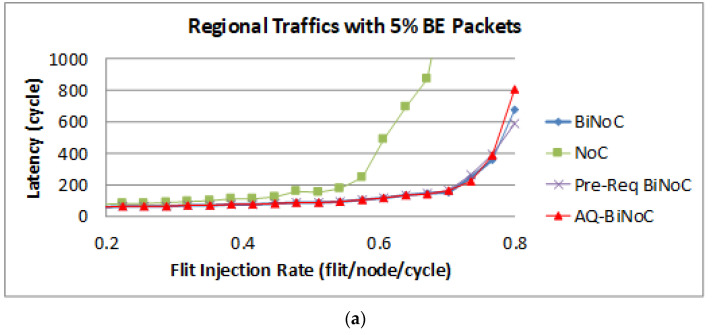

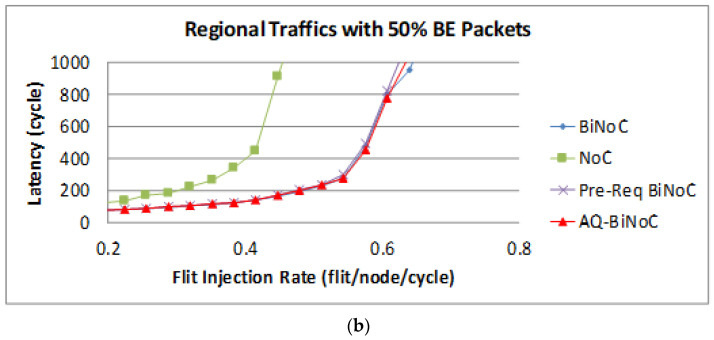
Packet latency analyses at (**a**) 5% and (**b**) 50% BE percentage under Regional Traffic.

The latency of BE packets is shown in Figure 19, and the improvement of the packets is not oblivious as GS packets shown in Figure 18 with the same reason for the close destinations in the tested regional traffics.

**Figure 20:**
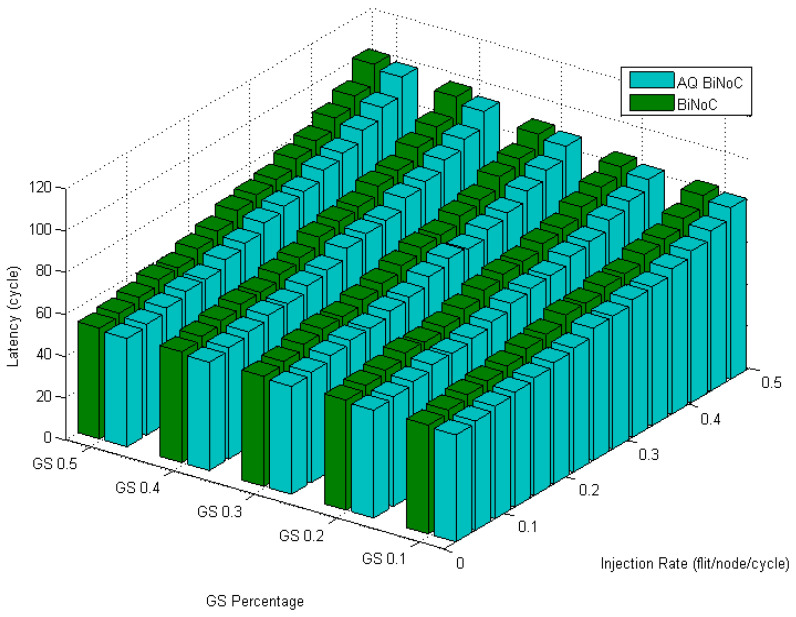
Latency of AQ-BiNoC compared with BiNoC in Regional Traffic.

Figure 20 displays the latencies in regional traffic of AQ-BiNoC compared with BiNoC. Even under the condition where the GS packet percentage and the injection rate are high, we can observe that the AQ-BiNoC has better latency performance than BiNoC and NoC architectures. The regional traffic sends packets to the destinations which are close to the source coordinates. The improvement is mainly provided by the inter-router channel arbitration functionality.

**Figure 21:**
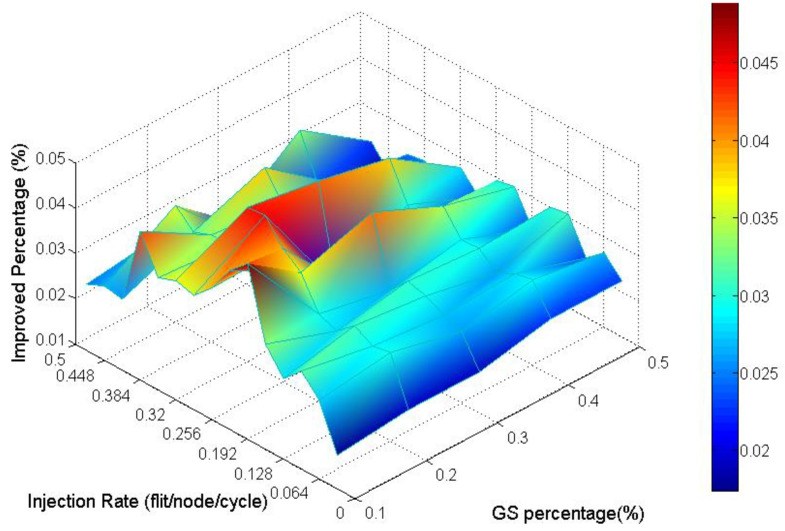
Performance improvement of AQ-BiNoC compared with BiNoC in Regional Traffic.

Analyzing the overall performance improvement of regional traffic as shown in Figure 21, we found that the improved percentage is generally more minor than it is in uniform traffic. But we can observe that under a higher GS packet percentage, the improvement still does not decay critically.

### 4.4. Experiments with Transpose Traffics

**Figure 22:**
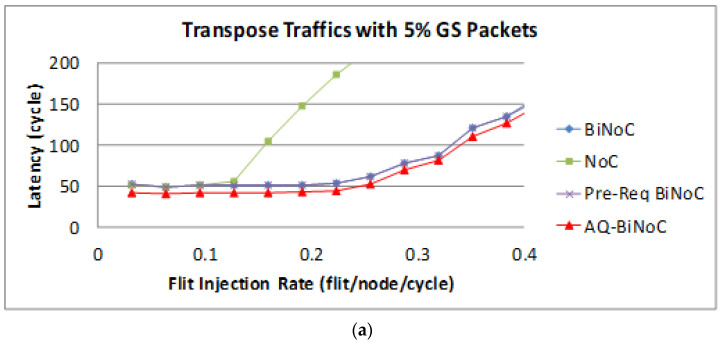

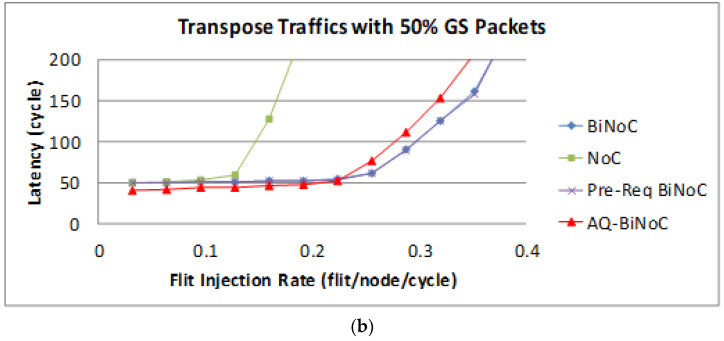
Packet latency analyses at (**a**) 5% and (**b**) 50% GS packet percentage under Transpose Traffic.

Figure 22a,b represent the GS packet latency at varying injection rates (from 0.032 to 0.4) with GS packet percentages of 5% (low) and 50% (high), respectively. As Figure 22a shows, we can discover that the average latency of AQ-BiNoC is lower than BiNoC, Pre-Req BiNoC, and traditional NoC in percentages of 11.84%, 11.44%, and 58.98%, respectively. Figure 22b shows the latency of regional traffic under a higher GS packet percentage of 50%, the average latency of AQ-BiNoC is lower than BiNoC, Pre-Req BiNoC, and traditional NoC in percentages of −20.98%, −23.35%, and 85.02%, respectively. As Figure 22b shows, the latency improvement is decreased as the GS packet percentage grows. The latency of AQ-BiNoC is worse than the original BiNoC as the latency grows up to twice the zero-load latency. The reason for this effect may be that since the transpose traffic causes congestion at router coordinate (*i*,*i*), the penetrative ability is limited by half of the network. Additionally, the anticipative bi-direction channel control is also useless because the transpose traffic data have only one direction to flow. 

**Figure 23:**
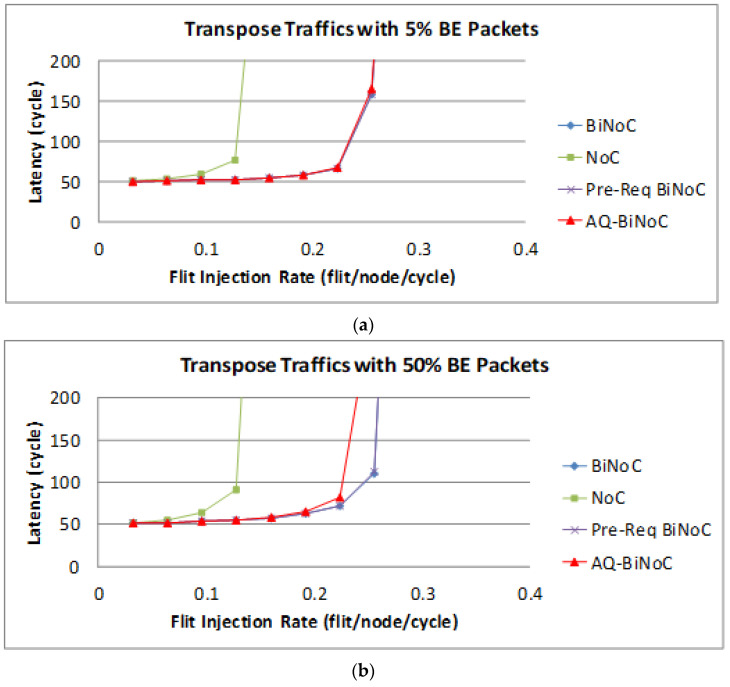
Packet latency analyses at (**a**) 5% and (**b**) 50% BE percentage under Transpose Traffic.

The latency of BE packets is shown in Figure 23, and the improvement of either packet is not as oblivious as GS packets shown in Figure 23a. By contrast, with a 50% BE percentage, Figure 23b shows that the latency of AQ-BiNoC is worse than the original BiNoC with the same reason discussed for Figure 23a. However, the negative impact could be minor since the performance worsens near the saturation point at a flit injection rate of 0.22.

**Figure 24:**
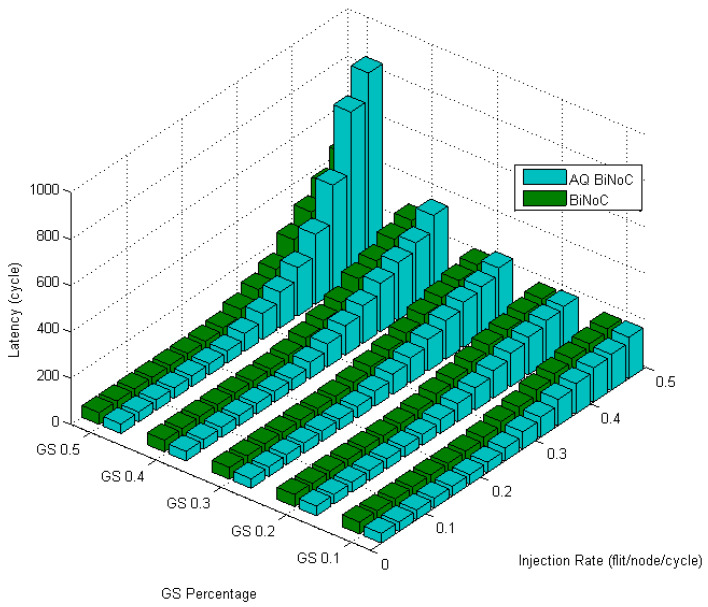
Latency of AQ-BiNoC compared with BiNoC in Transpose Traffic.

Figure 24 shows the latencies in transpose traffic of AQ-BiNoC compared with BiNoC. The performance becomes worse when the GS packet percentage and the injection rate are high, but AQ-BiNoC has better latency performance than BiNoC and NoC architectures under low injection rates. Since transpose traffic sends packets to the destinations in which the coordinate is the transpose value of the sources, most of the packet flow will be stuck and against each other at the routers whose coordinate is (*i*,*i*). Compared with BiNoC, the AQ-BiNoC router receives fewer sources; thus, the congestion will be more serious than the competition in BiNoC.

**Figure 25:**
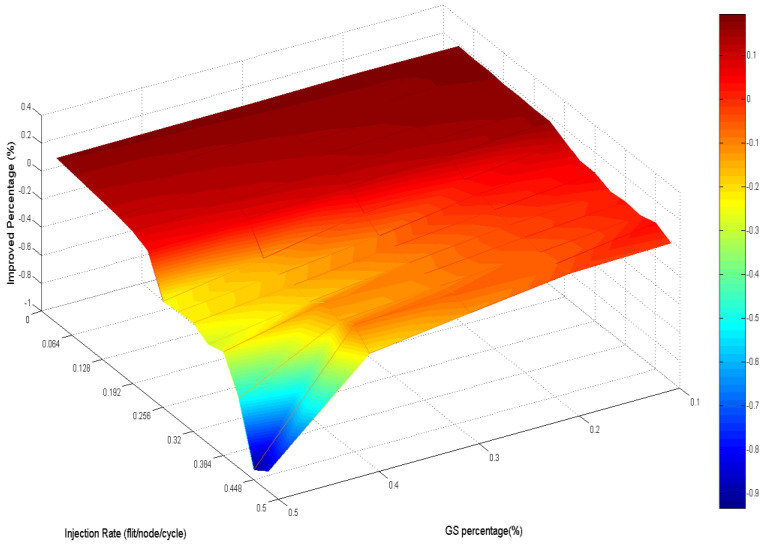
Performance improvement of AQ-BiNoC compared with BiNoC in Transpose Traffic.

Figure 25 emphasizes this phenomenon by the worse latency under high GS packet percentage and high injection rate.

### 4.5. Experiments with Hotspot Traffics

**Figure 26:**
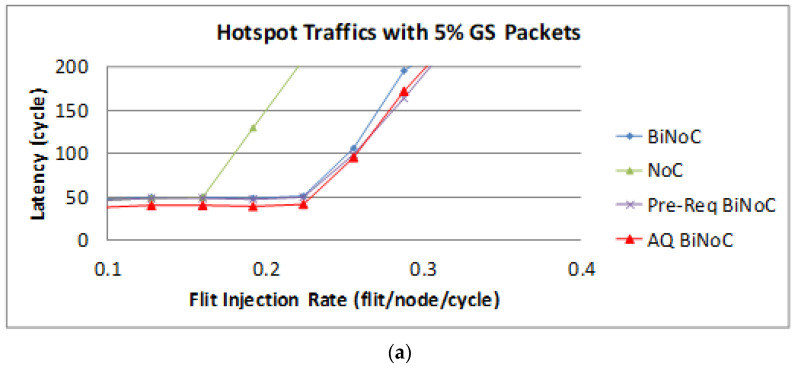

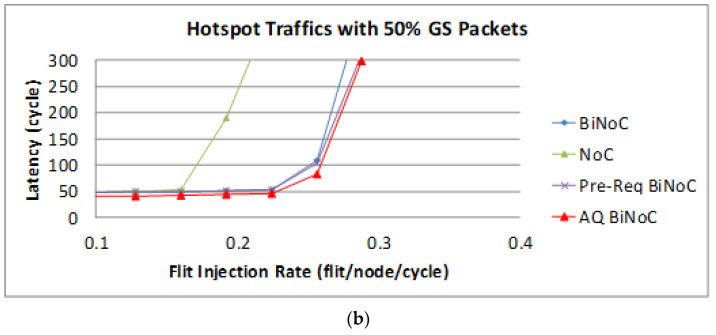
Packet latency analyses at (**a**) 5% and (**b**) 50% GS packet percentage under Hotspot Traffic.

Figure 26a,b represent the GS packet latency at varying injection rates (from 0.032 to 0.322) with GS packet percentages of 5% (low) and 50% (high), respectively. As Figure 26a shows, we can discover that the average latency of AQ-BiNoC is lower than BiNoC, Pre-Req BiNoC, and traditional NoC in percentages of 11.21%, 6.30%, and 50.06%, respectively. Figure 26b shows the latency of regional traffic under a higher GS packet percentage of 50%, the average latency of AQ-BiNoC is lower than BiNoC, Pre-Req BiNoC, and traditional NoC in percentages of 17.17%, 6.23%, and 68.35%, respectively. The performance saturation point of flit injection rate in hotspot traffic is worse than other traffic types because under high channel utilization at a small area, the channel bandwidth resources of local routers in the small area will be consumed soon. We can discover this under the burst traffics with Hotspot. AQ-BiNoC still received a considerable latency performance enhancement, even in the high GS packet percentage as shown in Figure 26b.

**Figure 27:**
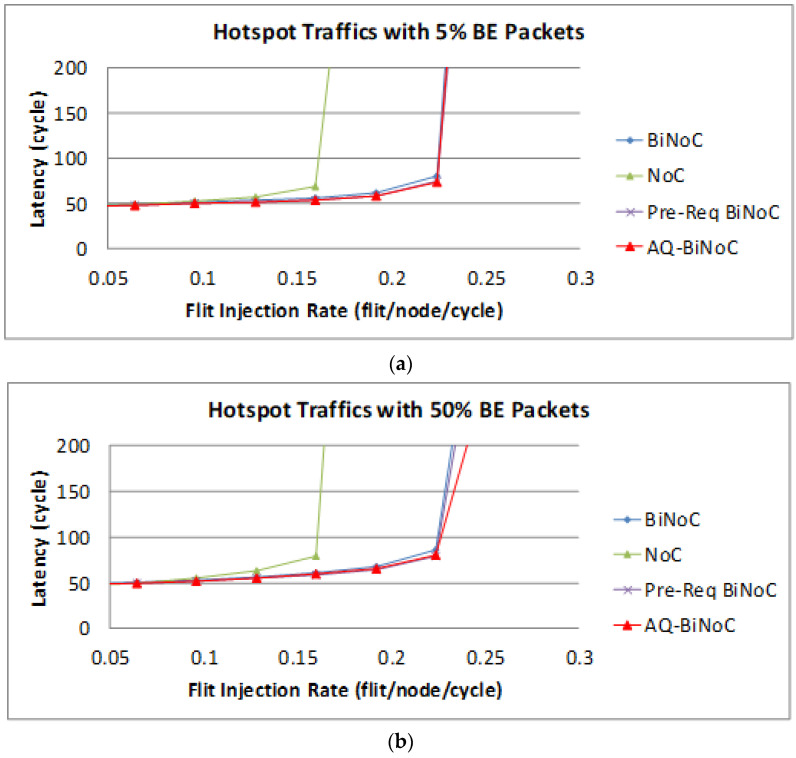
Packet latency analyses at (**a**) 5% and (**b**) 50% BE percentage under Hotspot Traffic.

The latency of BE packets is shown in Figure 27, AQ-BiNoC still had the best latency performance, especially in both low and high GS packet percentages.

**Figure 28:**
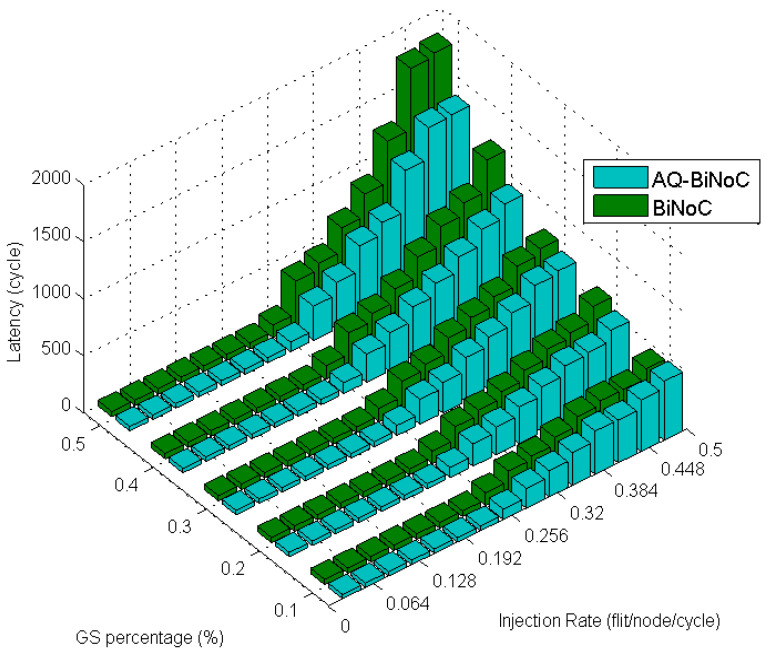
Latency of AQ-BiNoC compared with BiNoC in Hotspot Traffic.

Figure 28 reveals the latencies in hotspot traffic of AQ-BiNoC compared with BiNoC. The overall performance shows that our proposed AQ-BiNoC works well under such similarity to a real traffic case.

**Figure 29:**
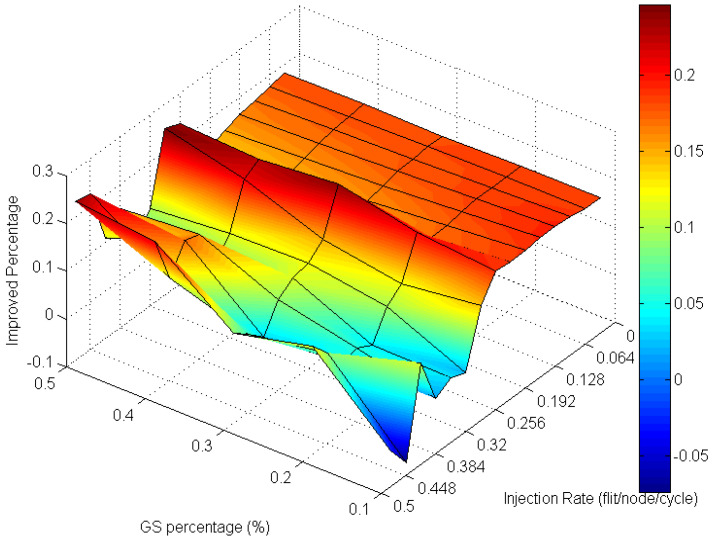
Performance improvement of AQ-BiNoC compared with BiNoC in Hotspot Traffic.

Figure 29 demonstrates the improved percentage of AQ-BiNoC compared with BiNoC. The performance decayed when the injection rate was high. However, in the overall situation, AQ-BiNoC has better latency.

### 4.6. Throughput Performance Analyses

Furthermore, in this section, we will evaluate the throughput performance of the proposed AQ-BiNoC scheme. The maximum throughput of an NoC can be analogous to the maximum-flow problem with multiple sources and sinks in graph algorithms [20]. Given a flow network graph *G* = (*V*, *E*), each edge (*u*, *v*) ∈ *E* and vertices *u*, *v* ∈ *V*. A cut *c*(*S*, *T*) of flows *f*(*S*, *T*) in a network *G* is a partition of *V* into *S* and *T* (*V* = *S* + *T*) such that *s* ∈ *S* and *t* ∈ *T*. A minimum cut of a flow network is a cut whose capacity is minimum overall cuts of the network. The max-flow-min-cut theorem tells us that a flow *f*(*s*, *t*) is maximum if and only if its residual network contains no augmenting path (i.e., *c*(*s*, *t*) is minimal). The throughput of a flow network *G* can be represented as:

(1) $$t\left(G\right)=f\left(S,T\right)=\underset{s\in S}{\sum }\underset{t\in T}{\sum }f\left(s,t\right)\leq \underset{s\in S}{\sum }\underset{t\in T}{\sum }c\left(s,t\right)=c\left(S,T\right)$$

And the maximal throughput of a flow network *G* equals the minimum cut:

(2) Max t(G)=Max f(S,T)=Min c(S,T)

**Figure 30:**
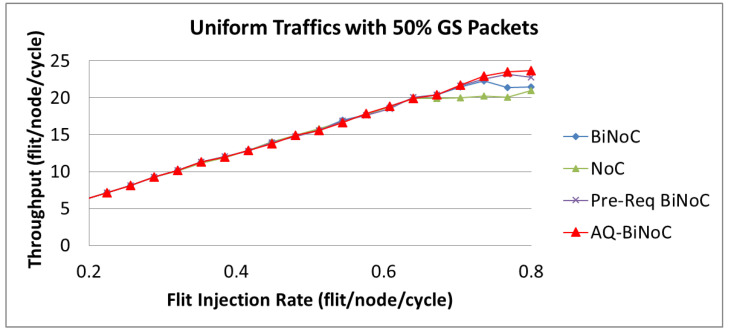
Packet throughput analyses at 50% GS packet percentage under Uniform Traffic.

A network might perform inconsistently on the merits of throughput and latency. Accordingly, another key challenge is to achieve the performance balance between latency and throughput. For QoS services, we focus on the analyses of 50% (high) GS packets. As demonstrated in Section 4.2, Section 4.3, Section 4.4 and Section 4.5, the proposed AQ-BiNoC scheme can improve the BiNoC performance in terms of packet latency, especially for the high-priority GS packet demanding QoS services in most traffic types. Although AQ-BiNoC is worse than the original BiNoC for the transpose traffic with 50% GS packets, it is reasonable that BiNoC is less efficient than NoC for the reason discussed in Section 4.4. Accordingly, except for the transpose traffic, we analyze the simulation results under the uniform, regional, and hotspot traffics, at varying injection rates (from 0.032 to 0.8) with GS packet percentages of 50%. As Figure 30 shows, the throughput performances with 50% GS packets of AQ-BiNoC, Pre-Req BiNoC, BiNoC, and traditional NoC are similar at lower flit injection rates. However, we can discover that AQ-BiNoC overcomes the other methods from the high flit injection rates of 0.7. 

**Figure 31:**
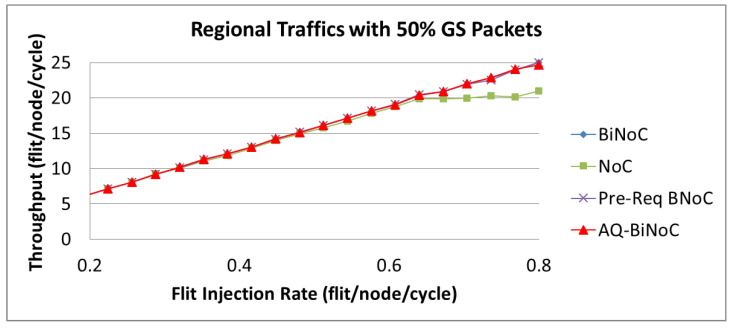
Packet throughput analyses at 50% GS packet percentage under Regional Traffic.

For regional traffics, as well as uniform traffic, AQ-BiNoC has a similar throughput performance compared with BiNoC with 50% GS packets as shown in Figure 3. This phenomenon is similar to the latency performance under regional traffic in Figure 18b, since the regional traffic is transmitting data to a close destination, it causes AQ-BiNoC, Pre-Req BiNoC, and BiNoC to have the similar performance curves as discussed in Section 4.3. However, they still overcome traditional NoC in the throughput performance with 50% GS packets as shown in Figure 31.

**Figure 32:**
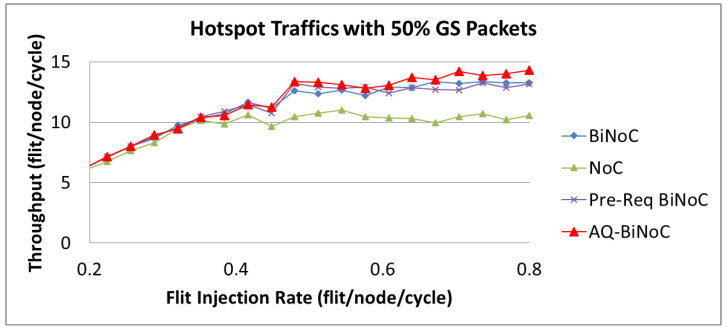
Packet throughput analyses at 50% GS packet percentage under Hotspot Traffic.

As Figure 32 shows, we can discover that the maximal throughput of hotspot traffics is around 15 flits/node/cycle, which is lower than that of uniform and regional traffics. It is because at a high flit injection rate with high channel utilization of regional traffic, channel bandwidth resources of local routers will be consumed soon. This phenomenon corresponds to the latency performance depicted in Figure 26b and discussed in Section 4.5. As Figure 32 shows, AQ-BiNoC still performs the best throughput performance at the high flit injection rate, especially under a higher GS packet percentage of 50%. 

As Figure 30, Figure 31 and Figure 32 show, the throughput enhancement of the proposed AQ-BiNoC is limited. The reason is that the maximal throughput limitation is relevant to the minimum cut of the flow network based on the max-flow-min-cut theorem as described in Equations (1) and (2). This phenomenon is especially noticeable in regular and local traffic flows, such as the regional traffic shown in Figure 31. However, in experiments with the uniform, regional, and hotspot traffics, AQ-BiNoC still performed the best in both merits of latency and throughput. For the purpose of further enhancing the QoS performance of the conventional QoS-aware BiNoC architecture [30], the proposed AQ-BiNoC can efficiently decrease the flit latency under a higher GS packet percentage of 50%, without sacrificing throughput performance in most traffic types and loads.

### 4.7. Implementation Overhead Analyses

As Table 2 lists, the four architectures were designed in Verilog, synthesized by Synopsys Design Compiler [33]. Compared with the existing method of BiNoC, the area, power, and timing overheads for implementing a router with the proposed AQ-BiNoC controls were as small as 5.01%, 1.44%, and 2.54%, respectively. However, as mentioned in the section of performance evaluations, for uniform, regional, and hotspot traffics with 5% (/50%) GS packets, it showed that the average latency of AQ-BiNoC is lower than BiNoC in percentages of 14.95% (/12.57%), 5.27% (/1.80%), and 11.21% (/17.17%), respectively. Moreover, the throughput performance of AQ-BiNoC is superior to BiNoC under a higher GS packet percentage of 50%. This demonstrated that the superiority of AQ-BiNoC is over existing methods of BiNoC, Pre-Req BiNoC, and traditional NoC.

## 5. Conclusions

In this paper, we proposed a novel self-reconfigurable network architecture AQ-BiNoC which features the use of anticipative QoS control with penetrative switch ability to improve the overall network performance, especially for high-priority packets. Experiments using four different featuring traffic patterns verified the network performance with different GS packet percentages under various packet injection rates. Experimental results demonstrate that the proposed AQ-BiNoC overcomes all of the other competitors of Typical-NoC, BiNoC, and Pre-Req BiNoC to perform less packet transmission latencies without sacrificing throughput performance in most traffic types and loads. Compared with the previous works resulting in packets stalled in the router pipeline caused by changing channel’s direction, the novel inter-router packet switching strategy of AQ-BiNoC, which enables the designed router to anticipate the channel conditions of the neighbor’s neighbor, can thus rapidly bypass the 50% high-priority packet in experiments to its destination for QoS services. This is intended to be the major contribution of this paper.

## Figures and Tables

**Table 1:** NoC router configurations used in the experiments.

Architecture	Typical-NoC	BiNoC	Pre-Req BiNoC	AQ-BiNoC
Total # VC *	4	4	4	4
Each VC * Size	8	8	8	8
Total Channels	5-in 5-out	10-inout	10-inout	10-inout
Channels/Dir.	1-in 1-out	2-inout	2-inout	2-inout
Each Buf. Size	32 flits	32 flits	32 flits	32 flits
Total Buf. Size	160 flits	160 flits	160 flits	160 flits
Crossbar	5 × 5	10 × 10	10 × 10	10 × 10

* VC: Virtual Channel.

**Table 2:** Area and Power Analyses.

Architecture	Typical-NoC	BiNoC	Pre-Req BiNoC	AQ-BiNoC
Area	369,743	448,010	457,994	470,490
Normalized Area	82.53%	100%	102.22%	105.01%
Power	42.5285 mw	42.9385 mw	43.0414 mw	43.5554 mw
Normalized Power	99.05%	100%	100.24%	101.44%
